# Supplementary material for: The transcriptomic landscape of monosomy X (45,X) during early human fetal and placental development
Source: Commun Biol. 2025 Feb 16;8:249. doi: 10.1038/s42003-025-07699-4 (PMC11830783; doi:10.1038/s42003-025-07699-4)
Supplement: Supplementary file 2 — Reporting summary [file 42003_2025_7699_MOESM2_ESM.pdf]

nature portfolio

Corresponding author(s): John Achermann

Last updated by author(s): Jan 18, 2025

Reporting Summary

Nature Portfolio wishes to improve the reproducibility of the work that we publish. This form provides structure for consistency and transparency in reporting. For further information on Nature Portfolio policies, see our Editorial Policies and the Editorial Policy Checklist.

Statistics

For all statistical analyses, confirm that the following items are present in the figure legend, table legend, main text, or Methods section.

n/a	Confirmed	
☐	☒	The exact sample size (*n*) for each experimental group/condition, given as a discrete number and unit of measurement
☐	☒	A statement on whether measurements were taken from distinct samples or whether the same sample was measured repeatedly
☐	☒	The statistical test(s) used AND whether they are one- or two-sided<br>*Only common tests should be described solely by name; describe more complex techniques in the Methods section.*
☐	☒	A description of all covariates tested
☐	☒	A description of any assumptions or corrections, such as tests of normality and adjustment for multiple comparisons
☐	☒	A full description of the statistical parameters including central tendency (e.g. means) or other basic estimates (e.g. regression coefficient) AND variation (e.g. standard deviation) or associated estimates of uncertainty (e.g. confidence intervals)
☐	☒	For null hypothesis testing, the test statistic (e.g. *F*, *t*, *r*) with confidence intervals, effect sizes, degrees of freedom and *P* value noted<br>*Give P values as exact values whenever suitable.*
☒	☐	For Bayesian analysis, information on the choice of priors and Markov chain Monte Carlo settings
☒	☐	For hierarchical and complex designs, identification of the appropriate level for tests and full reporting of outcomes
☒	☐	Estimates of effect sizes (e.g. Cohen's *d*, Pearson's *r*), indicating how they were calculated

Our web collection on statistics for biologists contains articles on many of the points above.

Software and code

Policy information about availability of computer code

Data collection	No software was used
Data analysis	Standard open source packages were used for analysis as outlined in the Methods. GraphPad (v9.5.1) was used for qRT-PCR analysis.

For manuscripts utilizing custom algorithms or software that are central to the research but not yet described in published literature, software must be made available to editors and reviewers. We strongly encourage code deposition in a community repository (e.g. GitHub). See the Nature Portfolio guidelines for submitting code & software for further information.

Data

Policy information about availability of data

All manuscripts must include a data availability statement. This statement should provide the following information, where applicable:

- Accession codes, unique identifiers, or web links for publicly available datasets
- A description of any restrictions on data availability
- For clinical datasets or third party data, please ensure that the statement adheres to our policy

Bulk RNA-sequencing data are deposited in ArrayExpress/Biostudies (accession number MTAB-13673).

Research involving human participants, their data, or biological material

Policy information about studies with human participants or human data. See also policy information about sex, gender (identity/presentation), and sexual orientation and race, ethnicity and racism.

Reporting on sex and gender	The whole purpose of this work is to compare tissues with different karyotypes (45,X; 46,XY; 46,XX). As this is fetal material, we use karyotype as a proxy for biological sex, and avoid terms such as "male" or "female". We do not use "gender" in a non-biological context. All groups were balanced in number and analysis, as this was the main focus of the study. The comparisons of different karyotypes.
Reporting on race, ethnicity, or other socially relevant groupings	We cannot report on race, ethnicity or social-economic variables as all tissues that are provided by the Human Developmental Biology Resource are pseudononymised, and these data are not available.
Population characteristics	The samples are all fetal tissue samples with very clearly defined developmental age and karyotype/sex.
Recruitment	Samples with a 45,X karyotype were collected sequentially, when available and consent provided. There was no selection bias in this process. The 46,XY and 46,XX samples were obtained randomly to match the developmental stage of the 45,X samples.
Ethics oversight	NRES London-Fulham (18/LO/0822) and Newcastle (18/NE/0290)

Note that full information on the approval of the study protocol must also be provided in the manuscript.

Field-specific reporting

Please select the one below that is the best fit for your research. If you are not sure, read the appropriate sections before making your selection.

☒ Life sciences ☐ Behavioural & social sciences ☐ Ecological, evolutionary & environmental sciences

For a reference copy of the document with all sections, see nature.com/documents/nr-reporting-summary-flat.pdf

Life sciences study design

All studies must disclose on these points even when the disclosure is negative.

Sample size	For bulk RNA seq studies, a minimum of 3 samples is usually considered standard for assessing differential gene expression. We were able to include groups of 4 samples in each comparison, and 6 samples for placenta. As differences between samples of the same tissue are somewhat limited it would of course have been even better to have had a larger numbers but this was not possible given the rarity of the 45,X tissues. In general we have been able to generate reasonable datasets of differential gene expression when adjusted for multiple comparisons and with adjusted-p<0.05. REVISED MANUSCRIPT In the revised manuscript, we have generated a "multi-tissue" group of n=20 for each three karyotypes (45,X; 46,XX and 46,XY; total n=60) to increase power, add used several different approaches to data analysis and presentation, as suggested by Reviewers. These approaches were useful although the final results changed little. All data are available in Supplementary files and in repositories. The study of confirmed placental mosaicism had the number of samples in the 45,X placenta increased to n=18, including sampling three placentae in four different sites. The description of sampling approaches has been expanded.
Data exclusions	No data were excluded.
Replication	Bulk RNA seq data were verified by 1) comparing 45,X and 46,XY and 45,X and 46,XX studies separately, and obtaining consistent findings for many genes across multiple tissues except for the obvious predicted differences due to Y chromosome or X inactivation genes. 2) qRT-PCR of key genes identified to replicate differences in OVCH1-AS1 and to verify no differences in 4 genes in the region. 3) Immunohistochemistry to analyze expression of CSF2RA at the protein level. REVISED MANUSCRIPT Several different approaches were taken to reanalyse the data, including a combined "multi-tissue" group, linear mixed methods analysis and correlation plots, and the original findings were validated further using their strategies.
Randomization	All 45,X samples that became available were included and control tissues were age matched on a random basis without any available insight in to other characteristics.
Blinding	Blinding was not relevant to this study and samples were included randomly based on availability.REVISED MANUSCRIPT Much more detailed methods are provided about sampling and operating pipelines.

Reporting for specific materials, systems and methods

We require information from authors about some types of materials, experimental systems and methods used in many studies. Here, indicate whether each material, system or method listed is relevant to your study. If you are not sure if a list item applies to your research, read the appropriate section before selecting a response.

Materials & experimental systems

n/a	Involved in the study
☐	☒ Antibodies
☒	☐ Eukaryotic cell lines
☒	☐ Palaeontology and archaeology
☒	☐ Animals and other organisms
☒	☐ Clinical data
☒	☐ Dual use research of concern
☒	☐ Plants

Methods

n/a	Involved in the study
☒	☐ ChIP-seq
☒	☐ Flow cytometry
☒	☐ MRI-based neuroimaging

Antibodies

Antibodies used

For immunohistochemistry - CSF2RA antibody Origene TA323990S primary rabbit polyclonal

Validation

1) The manufacturers data sheet (https://cdn.origene.com/datasheet/ta323990.pdf) shows specificity for the antibody on thyroid and cervical cancer samples that was abolished synthetic peptide.
2) Our department of histopathology optimised the antibody on independent placenta samples (based on human tissue atlas expression) and the results and protocols were independently and validated by an experienced histopathologist. 3) This was classed as a verified antibody on BenchSci.

Plants

Seed stocks

n/a

Novel plant genotypes

n/a

Authentication

n/a
